# A 10-Year Retrospective Review of the Histology of Symptomatic and Asymptomatic Unilateral Nasal Polyps

**DOI:** 10.7759/cureus.44526

**Published:** 2023-09-01

**Authors:** Augustus Diaz, Francis De Castro, Venkat Reddy, Kel Anyanwu

**Affiliations:** 1 Ear, Nose and Throat (ENT) Department, Royal Cornwall Hospital, Truro, GBR

**Keywords:** surgery, histology, nose, cancer, polyp, nasal

## Abstract

Background

The aim of this study was to determine the histological diagnosis of unilateral nasal polyps and to determine the prevalence of neoplastic pathologies. This study also assessed difference in pathologies whether patients presented symptomatically or were asymptomatic (if they had a mass found incidentally for unrelated throat symptoms).

Method

This was a 10-year retrospective study of patients undergoing unilateral nasal mass surgery between 2004 and 2014 at a UK district general hospital. We recorded patient demographics, laterality, histology, symptoms, clinical suspicion, and imaging findings.

Results

123 patients were included who underwent unilateral surgery between 2004 and 2014 (male n=83, female n=40; mean age 56 years ± 19.5). The majority were of inflammatory origin (n=92; 74.8%). The most common benign neoplastic cause was inverted papilloma (n=19; 15.4%). A number of malignant neoplastic causes were also found, including: melanoma (n=3; 2.44%), olfactory neuroblastoma (n=2; 1.63%), and other non-inflammatory masses (n=7; 5.69%). 15 of these masses were found incidentally, with 14 being inflammatory, and one an olfactory neuroblastoma; therefore, 6.67% of our incidental unilateral nasal masses were found to be of neoplastic pathology.

Conclusion

This study's findings support the continued practice of routine biopsy of unilateral nasal masses for histological diagnosis, irrespective of whether they are symptomatic or found incidentally. The accuracy of both clinical suspicion and radiological suspicion on CT scans is not adequate to alter this practice.

## Introduction

Nasal masses are a common finding in the otolaryngology department. These can often caused by nasal polyposis, which affects around 4% of the general population and this common condition presents as part of chronic rhinosinusitis with nasal polpyposis (CRSwNP) [1]. Unilateral nasal masses can also be a presentation of benign and malignant tumours, such as inverted papilloma, squamous cell carcinoma, adenocarcinoma, and olfactory neuroblastoma, as well as other non-inflammatory pathologies, such as sarcoidosis and fungal rhinosinusitis [2-5]. Nasal polyps are caused by mucosal tissue overgrowth of the nasal cavities and paranasal sinuses [2-5]. Patients may present with nasal obstruction, hyposmia or anosmia, facial pain or pressure, nasal discharge, epistaxis, sleep disturbance, or may be asymptomatic [3-5]. Whilst most patients present with symptoms, the routine use of fiber-optic nasal endoscopy (FNE) to assess the pharynx may result in the identification of incidental nasal polyps that cause no nasal symptoms. 

CRSwNP is treated with saline rinses and intranasal corticosteroids in the first instance and with oral corticosteroids if not responsive [5]. Functional endoscopic sinus surgery (FESS) can also be used [5]. Further investigation with nasendoscopy and CT imaging is indicated if there is no response to medical management or concern about a neoplastic cause [4,5]. Clinical examination and radiological findings can be inadequate at determining pathology, which has led to routine biopsy and histological analysis of unilateral nasal masses via FESS or surgical biopsy [4]. Histology allows us to identify any malignant or non-inflammatory pathologies; it is also increasingly being used to assess eosinophilic infiltration in CRSwNP, given the novel use of biologics in these cases [5]. The 2020 European Position Paper on Rhinosinusitis and Nasal Polyps (EPOS) guidelines do not specifically advise routine histological analysis in all cases of CRSwNP, though this does appear to be common practice [5]. Historically, there has been debate around whether to send all unilateral nasal specimens for histological analysis. However, our experience of current practice is in line with the literature to continue the practice of sending all unilateral masses for histological analysis [3,6,7,8].

## Materials and methods

We conducted a 10-year retrospective analysis of all unilateral nasal polypectomies conducted at our institution, which is a district general hospital in the UK. An information analyst queried the hospital information systems to identify all patients who underwent procedures that included the terms “nasal polyp”, “exploration under anaesthesia (EUA) nose and biopsy”, “nasal biopsy”, and “biopsy post-nasal space”. 

Clinical notes were then reviewed to identify cases that underwent unilateral nasal polypectomy or unilateral biopsy or excision of a nasal mass or lesion. Cases of bilateral procedures were excluded. 136 cases were identified. Thirteen cases were then excluded due to: absence of histology results or incorrect coding due to no masses being found intraoperatively.

Data was recorded on a Microsoft Excel (Microsoft Corp., Redmond, USA) spreadsheet, including patient demographics, laterality, nasal symptoms (if any) at presentation, clinical suspicion radiologically and intra-operatively, and histology findings. Histology findings were separated into inflammatory and neoplastic aetiologies. The symptomatic group included any patients with nasal symptoms including: nasal obstruction, rhinorrhea, hyposmia or anosmia, epistaxis, facial pressure, or pain. The incidental group excluded patients with any nasal symptoms and were patients who had incidentally found polyps on FNE for investigation of the pharynx.

Descriptive statistics were used to explore patient characteristics. Sensitivity, specificity, and positive and negative predictive values were calculated for operative clinical suspicion and radiological suspicion for both groups.

Statistical analysis was conducted using SPSS Statistics 22 (IBM Corp., Armonk, USA). Chi-square test was used to compare the incidental and non-incidental groups by the prevalence of inflammatory and non-inflammatory pathology. Binary logistic regression was performed considering nasal symptoms as independent variables and pathology as the dependent variable.

As this was a retrospective study, the institutional audit and clinical improvement department deemed that ethical approval was not required.

## Results

One hundred and twenty-three patients were included in our study who had histology results for unilateral nasal masses between 2004 and 2014. Our study consisted of 83 males (67.5%) and 40 females (32.5%), with a mean age of 56 years (range: 8-91 years ± 19.5). Laterality was even (62 right, 61 left). The symptomatic group had 108 patients whilst 15 were assigned to the incidental group. 

The results of histology are demonstrated in Table 1. Inflammatory polyps accounted for 74.8% of all unilateral nasal polyps and 25.2% of cases showed neoplastic pathologies. Within the asymptomatic/incidental group (n=15), there was one case of neoplastic pathology (6.7%), which was an olfactory neuroblastoma. In the symptomatic group (n=108), 30 cases were diagnosed as neoplastic pathologies (27.8%). The higher prevalence of neoplastic pathologies in the symptomatic group, compared with the incidental group was not statistically significant when compared with the chi-square test (p>0.05).

**Table 1:** Histology results of unilateral nasal specimens

Histology	Symptomatic	Incidental	Total
Inflammatory	78	14	92
Inverted papilloma	19	0	19
Malignant melanoma	3	0	3
Olfactory neuroblastoma	1	1	2
Other neoplastic causes	7	0	7
TOTAL	108	15	123

A review of operative notes indicated the operative clinical suspicion for whether the polyp was inflammatory or non-inflammatory in 112 of the 123 cases. The comparison with the final histology is demonstrated in Table 2. Operative clinical suspicion for identifying a non-inflammatory polyp demonstrated a sensitivity of 73.1%, a specificity of 87.2%, a positive predictive value of 63.3%, and a negative predictive value of 91.5%.

**Table 2:** Comparison of operative clinical suspicion with final histology (n=112)

	Histology	
Operative Suspicion	Inflammatory	Other pathology
Inflammatory	75	7
Other pathology	11	19

Pre-operative CT or MRI scan was conducted for 69 patients. A radiological suspicion was described for 57 patients. The comparison of radiological suspicion on CT sinuses with final histology is demonstrated in Table 3. Radiological suspicion for identifying a non-inflammatory polyp demonstrated a sensitivity of 53.8% and a specificity of 90.9%. The positive predictive value was 87.0%. The negative predictive value was 63.6%.

**Table 3:** Comparison of radiological suspicion for non-inflammatory pathology with final histology (n=57)

	Histology	
Radiological Suspicion	Inflammatory	Other pathology
Inflammatory	40	6
Other pathology	4	7

Considering the 108 patients who had symptomatic nasal polyps, the frequency of symptoms is detailed in Table 4. Some patients reported multiple symptoms. Binary logistic regression considering nasal symptoms as independent variables, and histology as the dependent variable did not demonstrate any symptom to be statistically significant in predicting non-inflammatory pathology (p>0.05).

**Table 4:** Symptoms recorded for unilateral polyp patients

Symptoms	Incidence (n)
Nasal obstruction	95
Rhinorrhea	32
Hyposmia/anosmia	28
Epistaxis/blood-stained rhinorrhea	20
Facial pressure/pain	15

## Discussion

This study has demonstrated the prevalence of neoplastic pathology in symptomatic and incidental nasal polyps with no statistically significant difference between the two. To our knowledge, this is the first study to differentiate between symptomatic and incidental, asymptomatic unilateral nasal polyps. The one neoplastic case in the incidental group was an olfactory neuroblastoma, with the remainder being inflammatory polyps. Inverted papilloma was the most common non-inflammatory pathology. Inverted papilloma is an important diagnosis as although inverted papilloma is benign, it can be locally invasive and have a tendency of recurrence. Furthermore, inverted papilloma has the potential for malignant transformation (usually to squamous cell carcinoma) in around 10% of cases [9]. Whilst the management of inverted papilloma still requires surgical resection, these patients need closer follow up post-operatively given its rate of recurrence of around 14% [9,10].

Studies of histology samples from suspected bilateral nasal polypectomies demonstrate they are almost always of inflammatory origin, however, there is a small chance of incorrect clinical diagnoses with non-inflammatory pathologies being found [1,6,8,11]. Asimakopoulos et al and Yeh et al recommended against routine histology for bilateral nasal polyps [1,8]. Garavello et al suggested that a cost-efficacy analysis was necessary [11]. Wong et al demonstrated non-inflammatory pathology in just 0.56%, in a systematic review covering 3772 bilateral nasal polyp cases but felt this was not sufficient to alter the current practice of sending all samples for histology for bilateral polyposis despite accepting that this was “low yield” [6].

Seven studies were identified that included the histology specifically of unilateral polyps (Table 5) [3, 7, 8, 12-15]. Although Romashko et al reported no cases of non-inflammatory pathologies, the remaining papers had an average prevalence between 11.6% and 27.3% [7]. There was generally not a significant correlation between symptoms and diagnosis from these studies. 

**Table 5:** Comparison of histology rates in studies of unilateral nasal polyps

Lead Author	Patients	Inflammatory pathology	Neoplastic	% of Non-inflammatory Pathologies
Asimakopolous [8]	23	18	5	21.7%
Kucur [12]	73	57	16	21.9%
Nair [13]	53	44	9	16.9%
Romashko [7]	13	13	0	0.0%
Shah [3]	69	61	8	11.6%
Tritt [14]	44	32	12	27.3%
Yaman [15]	32	25	7	21.9%

Only one paper assessing unilateral nasal polyposis gave a clinical suspicion rate, which was 85.5% accurate [2]. This highlights the risk of not sending specimens for routine pathology as suspected inflammatory polyps were shown to have malignant pathologies including squamous cell carcinoma, adenoid cystic carcinoma, and rhabdomyosarcoma [2]. Our study also explored whether specific symptoms could be predictive of sinister pathologies. Features suggestive of sinister polyps include: bleeding or epistaxis, pain, induration, neurological symptoms, and total nasal obstruction [2,5,16]. However, our data did not demonstrate any nasal symptoms to be statistically significant in doing so. Although Tritt et al identified a correlation between unilateral epistaxis and inverted papilloma, the remaining literature did not find a correlation between symptoms and pathology [14]. The lack of predictive symptoms and accuracy in clinical suspicion in unilateral polyposis warrants the use of routine histological analysis to avoid the consequences of a potentially missed sinister diagnosis. A relatively recent study from the USA found that the cost of a delayed or missed diagnosis was significantly outweighed by the cost of routine histological analysis [16]. A study in the Netherlands estimated histological analysis only accounted for around 1% of the overall cost of endoscopic removal of a polyp or sinus mass [17]. 

The limitations of the study arise from its retrospective nature. There was also a paucity of CT/MRI scanning data, which most likely reflects the time period of the study group and a lack of recording due to the integration of IT systems. CT imaging is now a standardised practice [5]. Furthermore, it is likely that other cases of unilateral nasal polyps might have been identified in outpatients but not subjected to biopsy, so the true number of unilateral nasal polyps diagnosed in the study period is not known. 

## Conclusions

This study's findings support the continued practice of routine biopsy of unilateral nasal polyps for histological diagnosis, irrespective of whether they are symptomatic or found incidentally, given the risk of neoplasm. The accuracy of both clinical suspicion and radiological suspicion on CT scans is not adequate to alter this practice. There is also further benefit in analysing histology given the development of novel biologic therapies. 
